# Living Liver Donor Paired Exchange: Can Anaesthesia Management Challenge?

**DOI:** 10.4274/TJAR.2025.241728

**Published:** 2025-02-11

**Authors:** Mehmet Ali Erdoğan, Muharrem Uçar, Yusuf Ziya Çolak, Duygu Demiröz, Oya Olcay Özdeş

**Affiliations:** 1 İstanbul Medeniyet University Faculty of Medicine, Department of Anaesthesia and Reanimation, İstanbul, Türkiye; 2 İnönü University Faculty of Medicine, Department of Anaesthesia and Reanimation, Malatya, Türkiye; 3 Battalgazi State Hospital, Clinic of Anaesthesia and Reanimation, Malatya, Türkiye

**Keywords:** Anaesthesia management, five living-donor, living-donor liver paired exchange, transplant anaesthesia, transplantation

## Dear editor,

Living-donor liver paired exchange (LPE) is a n effective strategy to increase the availability of organs for liver transplantation in patients with end-stage liver disease. Reasons for LPE include ABO incompatibility, inadequate hepatic mass, poor graft quality, anatomical considerations of the liver, and low residual liver volume in the donor.

It was reported that the first five-way [involving five living-donor hepatectomies and five living-donor liver transplants (LDLT)], paired exchange transplantation was performed simultaneously. The anesthetic challenges of this choice were also discussed.

We have shown that LPE can be performed at high-volume transplant institutes like ours, and is a useful strategy for ABO incompatibility and size incompatibility. Performing multiple LPEs simultaneously may present challenges in anesthesia management, and require a high-capacity center.

LPE offers a promising strategy to increase the organ supply for liver transplantation.^1^ This report describes the first-ever simultaneous five-way LPE transplantation (involving five living-donor hepatectomies and five LDLT). The anesthetic challenges associated with this complex process are also discussed.

Following thorough psychosocial evaluation, by psychiatrists to ensure suitability, written informed consent was obtained from all participating donors and recipients for the paired exchange program. The Liver Transplant Institute of İnönü University’s established matching algorithm facilitated the pairing process.^2^ Standard anesthesia monitoring protocols were employed for all donors, including a 3-lead electrocardiogram, oxygen saturation, non-invasive blood pressure, and bispectral index, before induction. Induction typically involved propofol (2.5 mg kg^-1^), fentanyl (1-2 µg kg^-1^), and rocuronium for muscle relaxation. Following intubation, vital signs (blood pressure and urine output), body temperature, and bleeding were closely monitored. Anesthesia was maintained with a combination of 1 MAC sevoflurane and remifentanil infusions. Restrictive fluid management was implemented until graft removal. After successful liver retrieval, aggressive fluid resuscitation was administered to restore the donor’s normovolemic state.

Multimodal postoperative analgesia was administered to donors including a transversus abdominis plane block and/or local anesthesia at the wound site, combined with intravenous morphine and dexketoprofen. Standard monitoring of the recipients included electrocardiography, pulse oximetry, capnography, temperature, urine output, coagulation profile, invasive arterial pressure, and central venous pressure (measured from the right internal jugular vein). The anesthesia management protocol for all recipients involved induction with propofol (1-2 mg kg^-1^) and fentanyl (1-2 µg kg^-1^), muscle relaxation with rocuronium (0.6-1 mg kg^-1^), and maintenance with sevoflurane (0.8-1 MAC), remifentanil, and rocuronium (as needed). Goal-directed fluid therapy with balanced saline solution and albumin supplementation was provided as needed. PVi (pleth variability index), Perfusion Index and Plethysmograph waveform analysis (Radical 7 Pulse CO-Oximeter™, Masimo) was used for goal-directed fluid therapy and cardiac ouput monitoring technique. The team minimized fluid administration to avoid dilutional coagulopathy. Red blood cell transfusions were administered if hemoglobin dropped below 8 g dL^-1^. Vasopressors were used for hypotension unresponsive to fluid resuscitation. Coagulation management was guided by Rotem.

In our institute, the annual case number of adult LDLT is 204, and the number of pediatric LDLT is 37. Our institute boasts eight experienced surgeons dedicated to donor procedures and another eight for recipient surgeries. Each senior surgeon was assisted by two fellow trainees. The anesthesia team comprised five senior anesthesiologists, each responsible for one donor-recipient pair. Additionally, two junior fellows worked under the supervision of senior anesthesiologists, assisting in either the donor or recipient operating room. Importantly, both surgical procedures and anesthesia management for all donors and recipients proceeded uneventfully, with no major complications observed.

**Figure 1:**
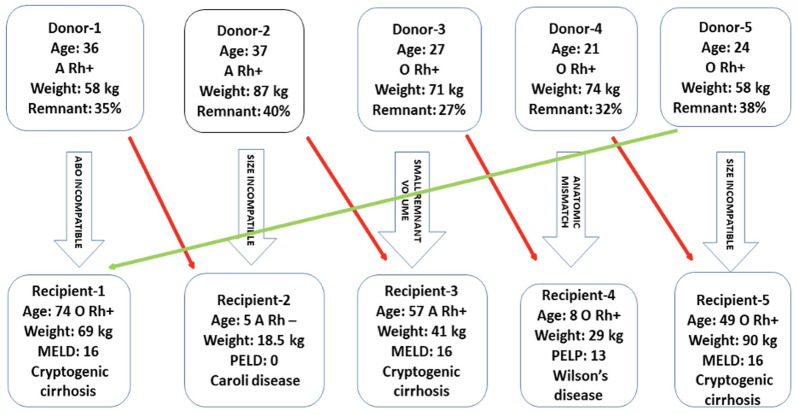
Diagrammatic representation of liver paired exchange

Pair-1 faced ABO incompatibility while the remaining pairs were incompatible in size. For Pair-5, the donor’s right liver lobe was considered too small due to a graft-to-recipient weight ratio of only 0.66%. In Pair-3, a right lobe hepatectomy was not feasible due to the recipient’s low residual liver volume (27%), and the left lobe was also deemed unsuitable due to its small size. Similarly, for the pediatric recipients in Pair-2 and Pair-4, the donor grafts were too large, and left lobe hepatectomy was not an option due to the presence of three arteries less than 1 mm in diameter in each patient. Figure 1 illustrates the details of this five-way LPE.

LPE offers a solution for situations where conventional LDLT is limited by factors such as small graft size, unsuitable recipient anatomy, specific donor anatomical features, and ABO incompatibility. LPE expands the pool of potential donors, benefiting patients who would otherwise have no chance of receiving a suitable deceased donor liver graft. Hepatic size incompatibility alone excludes 4-14% of potential LDLT donors. Gunabushanam et al.^3^ highlighted the effectiveness of LPE in expanding the donor pool and reducing waitlist mortality for liver transplant recipients. In our specific case, both ABO and size incompatibility necessitated LPE. Without this approach, none of these recipients would have been able to undergo transplantation.

The timing of pairing in LPE is crucial. While some centers perform donor and recipient surgeries simultaneously, others schedule them sequentially. To avoid complications associated with simultaneous LPE surgeries, stringent criteria are often established for donor and recipient selection.^4^ Given these considerations, we opted to perform the surgeries in this five-way LPE simultaneously.

Previous studies have documented the logistical complexities associated with performing LPE surgeries simultaneously. These challenges encompass the matching program, multi-site coordination of large teams (including senior surgeons, anesthesiologists, technicians, and nurses), and ensuring sufficient blood and blood product supplies.^1, 5^ To overcome these hurdles, we established ten distinct teams. Each team comprised experienced anesthesiologists, surgeons, and anesthesia technicians (all with over 20 years of LDLT experience) and was allocated a significant portion of hospital and operating room resources.

All donor operations and anesthesia inductions were initiated simultaneously to mitigate the risk of donor withdrawal. Any complications arising during anesthesia management, such as airway issues or invasive procedures, could lead to the cancellation of the entire LPE sequence, placing immense stress on the anesthesiologists.

Our experience demonstrates the feasibility of performing high-volume LPE at our transplant institute, highlighting its effectiveness in addressing ABO and size incompatibility issues. However, it is important to acknowledge that conducting multiple LPE surgeries simultaneously can pose significant challenges for anesthesia management and necessitates a high-capacity medical center. In addition, the main requirements for the anesthesiology team during the perioperative period are adequate infrastructure, experience, and close coordination with the surgeon and other stakeholders (blood center, nurse, technician).

## References

[1] Agrawal D, Saigal S, Jadaun SS, et al (2022). Paired exchange living donor liver transplantation: a nine-year experience from North India.. Transplantation.

[2] Yilmaz S, Sonmez T, Unver MU, et al (2023). The first 4-way liver paired exchange from an interdisciplinary collaboration between health care professionals and design economists.. Am J Transplant.

[3] Gunabushanam V, Ganesh S, Soltys K, et al (2022). Increasing living donor liver transplantation using liver paired exchange.. J Am Coll Surg.

[4] Hwang S, Lee SG, Moon DB, et al (2010). Exchange living donor liver transplantation to overcome ABO incompatibility in adult patients.. Liver Transpl.

[5] Lo AL, Sonnenberg EM, Abt PL (2019). Evolving swaps in transplantation: global exchange, vouchers, liver, and trans-organ paired exchange.. Curr Opin Organ Transplant.

